# The Dynamic Work study: study protocol of a cluster randomized controlled trial of an occupational health intervention aimed at reducing sitting time in office workers

**DOI:** 10.1186/s12889-019-6467-0

**Published:** 2019-02-13

**Authors:** Judith G. M. Jelsma, Lidewij R. Renaud, Maaike A. Huysmans, Jennifer K. Coffeng, Anne Loyen, Femke van Nassau, Judith E. Bosmans, Erwin M. Speklé, Allard J. van der Beek, Hidde P. van der Ploeg

**Affiliations:** 1 Department of Public and Occupational Health, Amsterdam Public Health research institute, Amsterdam UMC, Vrije Universiteit Amsterdam, Van der Boechorststraat 7, 1081BT Amsterdam, The Netherlands; 2 Department of Health Sciences, Faculty of Earth & Life Sciences, Amsterdam Public Health research institute, Amsterdam UMC, Vrije Universiteit Amsterdam, Amsterdam, The Netherlands; 3 Arbo Unie, Occupational Health Service, Utrecht, The Netherlands

**Keywords:** Sitting time, Office workers, Sit-stand desk, Dynamic workstation, Workplace, Randomized controlled trial, Physical activity, Cost-effectiveness

## Abstract

**Background:**

Large volumes of sitting time have been associated with multiple health risks. To reduce sitting time of office workers working for a Dutch insurance company, the Dynamic Work intervention was developed. The primary objective of this paper is to describe the study protocol of the Dynamic Work study, which aims to evaluate if this multicomponent intervention is (cost-)effective in reducing total sitting time on the short-term (≈3 months) and longer-term (≈12 months) compared to usual practice.

**Methods/design:**

This two-arm cluster randomized controlled trial will recruit 250 desk-based office workers working at different locations of an insurance company in the Netherlands. After baseline measurements, departments will be matched in pairs and each pair will be randomly assigned to the control or intervention condition. The multicomponent intervention contains organizational (i.e. face to face session with the head of the department), work environmental (i.e. the introduction of sit-stand desks and cycling workstations), and individual elements (i.e. counselling and activity/sitting tracker with a self-help program booklet). The counselling involves two group intervention sessions and four on-site department consultations with an occupational physiotherapist. Sitting time (primary outcome), upright time and step counts will be assessed objectively using the activPAL activity monitor at baseline, short-term (approximately 3 months) and longer-term (12 months). Other outcomes will include: self-reported lifestyle behaviours, anthropometrics, work-related outcomes (i.e. absenteeism, presenteeism, work performance, work-related stress), health-related outcomes (i.e. vitality, musculoskeletal symptoms, need for recovery, quality of life), and costs from both company and societal perspective. The study will include economic and process evaluations.

**Discussion:**

This study will assess the longer-term (cost-) effectiveness of a multicomponent workplace intervention aimed at reducing sitting time in comparison with usual practice. Furthermore, the process evaluation will provide insights in factors associated with successful implementation of this intervention.

**Trial registration:**

ClinicalTrials.gov NCT03115645; Registered 13 April 2017. Retrospectively registered.

## Background

Large volumes of sitting time have been associated with mortality and health problems, such as diabetes and cardiovascular disease [1–3]. Study results suggest that sitting more than 7–8 h per day is associated with increased all-cause and cardiovascular mortality rates [2, 3]. Although high levels of physical activity appear to attenuate these relationships, only levels in excess of five times the World Health Organization (WHO) physical activity recommendation might eliminate the health risks of sitting time [3].

Desk-based work is a likely cause for accumulating high levels of sitting. According to the recent Eurobarometer survey, white collar workers are likely to sit more than 7.5 h per day [4]. At the same time, most leisure time is also spent sitting, namely 85–90% of total leisure time in Australian and Dutch adults [5, 6]. Hence, occupational and leisure sitting time are primary targets for interventions aimed at reducing sitting time, especially in desk-based workers.

Sit-stand workstations and activity permissive workstations have shown promise as intervention tools to reduce sitting time at work [7–9] without work productivity loss. However, the majority of studies to date were pilot studies with only short-term follow-up. It has been reported that studies offering a behaviour change program in addition to the provision of such workstations (multicomponent intervention) might be more effective than solely providing the workstation [10, 11]. A recent larger scale study has shown sustained longer-term effects on workplace and overall daily sitting time by usage of a sit-stand workstation as part of an occupational behaviour change intervention [10].

Furthermore, activity/sitting trackers are promising tools to allow office workers to monitor their sitting time, which are well known tools to effectively increase physical activity levels in intervention studies [12]. Activity/sitting trackers have the potential to provide focus on the whole day, by providing feedback both during and outside working hours. The current study is one of the first to test a newly developed activity/sitting tracker integrated in a workplace intervention program. The tracker has been shown to be feasible [13] and valid [14] for use in physical activity and sedentary time intervention programs.

There is a need for sufficiently powered high quality studies that investigate longer-term effects of workplace interventions aimed at reducing total sitting time [7, 9, 15]. Therefore, in a Dutch insurance company the Dynamic Work intervention will be implemented to reduce sitting time during and outside work in office workers. This multicomponent intervention targets the organizational (i.e. management support), the work environmental (i.e. sit-stand desks and cycling workstations), and individual level (i.e. group health coaching and activity/sitting tracker). Occupational health interventions have the potential to be funded by the employer itself. Decisions about investment in health interventions are not only guided by the effectiveness of these programs, but also by the intervention costs and possible cost-savings (i.e. health care costs and productivity loss) as a consequence of the intervention (i.e. cost-effectiveness). Therefore, the primary objective of this paper is to present the study protocol of the Dynamic Work study, which aims to evaluate the (cost-)effectiveness of the multicomponent workplace intervention on the short-term (≈3 months) and longer-term (≈12 months).

## Methods

### Study design

**Fig. 1:**
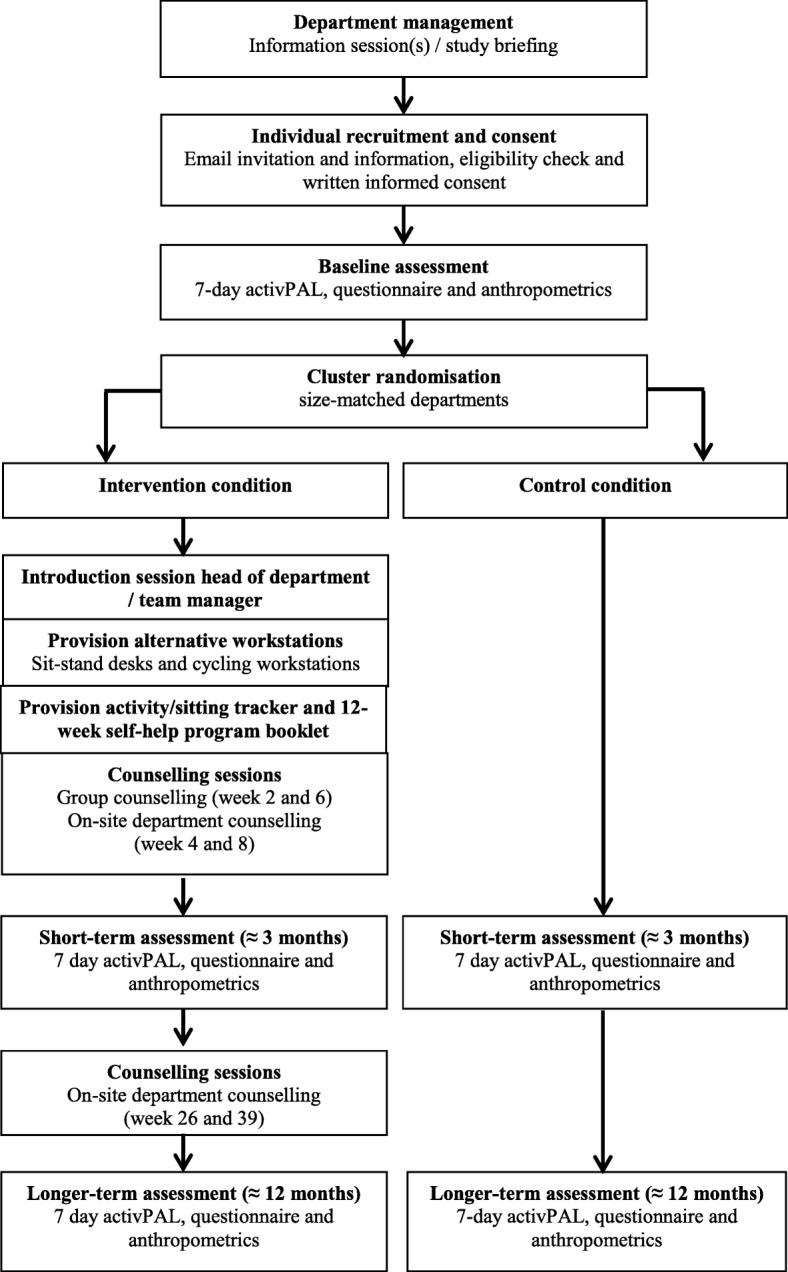
Study overview

This study is a size matched-pair cluster randomised controlled trial to investigate the effects of the Dynamic Work intervention on sitting time of office workers in the Netherlands. Different departments (clusters) of an insurance company in the Netherlands will be recruited and office workers of these departments will be asked to participate in this study. Departments will be matched based on size and work content and randomised into an intervention or control condition after baseline measurements. Cluster randomisation overcomes the problem of contamination between the intervention arms due to environmental changes on the work floor. Follow-up measurements will occur at approximately three months and twelve months after baseline measurements. A study overview showing the main components and timeline is given in Fig. 1. The study has been approved by the Medical Ethics Review Committee of the VU University Medical Center Amsterdam (2016.533). The trial sponsor is the VU University Medical Center Amsterdam. The study protocol has been registered at ClinicalTrials.gov Protocol Registration and Results System (NCT03115645).

### Setting

This study is targeting office workers of an insurance company that consists of around 14,000 permanent staff and 3000 casual staff on five locations in the Netherlands (Apeldoorn, Leeuwarden, Leusden, Tilburg and Zwolle). Employees are working in open-space offices without fixed desks and have the opportunity to work at home for 1 or 2 days a week. Work tasks comprise mostly computer-based work, but also involve meetings and traveling to meet with customers. Since 2014, the insurance company has a specialized department (healthy work) that is responsible for the vitality and health of all employees and runs a variety of health promotion programs.

### Participants

Office workers who are currently working for one of the recruited departments at the Dutch insurance company with an (intended) end date for employment until after the study has been completed are eligible to participate in this study. Office workers will be excluded from the study if he or she: 1) has a contract for less than 0.6 full time equivalent hours (FTE); 2) has a condition that could affect use of the intervention (e.g. wheelchair bound); 3) is pregnant.

### Recruitment

#### Recruitment of departments

Departments of a large Dutch insurance company that were not involved in a previous implementation study of sit-stand desks and cycling workstations will be eligible to participate. The head of the occupational health department of the insurance company will approach the managers of eligible departments. In an information session for the whole team or for the team managers, the study requirements and intervention will be outlined.

#### Recruitment of participants

Office workers of participating departments will be invited by email to participate in this study. Interested office workers can reply by email and need to answer eligibility questions. When found eligible, they will be invited for baseline measurements. Prior to the measurements, study requirements will again be explained and written informed consent will be obtained. Participation will be voluntary and participants can withdraw at any time without giving any reason. During the recruitment phase, the research team is in close contact with team managers of the participating departments and will attend team meetings (if possible) to boost recruitment if needed.

### Randomisation

Departments are the unit of randomisation, and they will be matched primarily on number of office workers and similarity of work content and tasks. Departments will be randomized into an intervention condition, receiving the Dynamic Work intervention on top of usual practice and a control condition, receiving usual practice only. Possible contamination of the control condition will be avoided as much as possible (e.g. not working on the same floor and not interacting too much with departments in intervention condition on the same or different locations). After baseline data collection, cluster randomisation using a list randomizer (www.random.org) will be performed by a researcher not involved in recruitment or data collection. The result of the randomisation will be shared with the head of the occupational health department of the insurance company, who will inform the heads of the departments and the office workers of the departments.

### Blinding

Since the baseline assessment will take place before randomization, participants and field work staff will be unaware of group allocation at baseline. After baseline, due to the nature of the intervention it will not be possible to blind participants. However, field work staff that perform the follow-up assessments will be kept blinded to group allocation. Researchers that process and clean the data will also be blinded to group allocation.

### Intervention

Both the intervention and control condition will receive usual practice, which includes prompting staircase use, suggesting and prompting walking routes and 5–10 min telephone routes with footsteps on the floor, and offering availability of lunch bags to take along on a lunch walk. In addition, the intervention condition will receive the Dynamic Work intervention, which is a multicomponent intervention containing organisational, environmental (i.e. sit-stand desks and cycling workstations) and individual elements (i.e. coaching, activity/sitting tracker with self-help program booklet), aimed to reduce sitting time during and outside work.

#### Organisational components

An occupational physiotherapist contacts the heads of the departments randomised into the intervention condition to plan an appointment for a face-to-face session. This face-to-face session is intended to provide information about the Dynamic Work intervention, to discuss expectations from the head of the department and plan the first team meeting to inform office workers of the department. Furthermore, organisational structures and processes important for study implementation are discussed (e.g. placement of intervention materials).

#### Environmental components

Sit-stand desks (VEPA, Hoogeveen, the Netherlands) and cycling workstations will be placed at the departments. The sit-stand desks allow office workers to alternate between sitting and standing, and can be adjusted electronically in height. Sit-stand desks will be placed in a 1 in 3 ratio with regular desks, with a minimum of four sit-stand desks at a department. All participating departments have flexible workplaces (i.e. no one has a fixed workplace). Resistance adjustable cycling workstations allow participants to cycle gently at a sit-stand desk which is in the standing position, (i.e. one cycling workstation will be placed per six participants). Each department will also receive a minimum of two office balls (vluvstuf, Breda, the Netherlands) which intend to make sitting more physically demanding, as the activity of the abdominal, back, leg and thigh muscles is higher to remain in an upright position and maintain balance compared to sitting in a regular office chair. The proper use of all these environmental elements of the Dynamic Work intervention will be explained by an occupational physiotherapist during the first team meeting at each department.

#### Individual elements

Individual level support will be provided by three occupational physiotherapists, trained in occupational health and wellness, during two team meetings of 30 min and during four on-site department counselling sessions. The first team meeting will be scheduled shortly after placement of the sit-stand desks and cycling workstations (≈week 2), followed by a second team meeting in week 6. On-site department counselling sessions will take place in week 4, week 8, week 26 and week 39.

In the first team meeting the occupational physiotherapist will discuss some broad knowledge about health risks of prolonged sitting and benefits of reducing sitting time and increasing standing and physical activity time. The proper use of the sit-stand desks and cycling workstations will be explained, the activity/sitting tracker “Activator” and self-help program booklet (which is handed out prior to this meeting) will be explained, and personalized behaviour change goals will be set.

During the second team meeting, the occupational physiotherapist will focus on: 1) the previous month’s experiences with the sit-stand desks, cycling workstations, Activator and self-help program booklet; 2) identifying and overcoming barriers in reducing sitting time and increasing standing and physical activity both during and outside work; and 3) behaviour change goal setting.

The on-site 30 min department counselling sessions are intended to reflect on the use of the workstations, answer individual questions from office workers (e.g. regarding ergonomic position, adjustment of workstations, recommendations for sitting, standing and physical activity, or personal goals, and dealing with barriers) and provide tips. The occupational physiotherapist will visit the department on multiple days (at least two) during the week and will shortly (5–10 min) attend a team meeting before the on-site department visit.

The Activator (PAL technologies, Glasgow, UK) is an activity/sitting tracker that allows traditional self-monitoring of steps as well as upright time (presented in min/day) and sitting time. Sitting time is presented as 1) percentage of wear time spent sitting per day, and 2) percentage of time spent sitting the last hour). It is worn on the front of the thigh, either in a pants pocket or attached with an elastic band to clothing covering the upper thigh (e.g. trousers, jeans, shorts, leggings, tights, dresses). The Activator provides feedback through a smartphone app via Bluetooth connection. It provides real time feedback and a seven day historical view of step counts, upright time and percentage of time spent sitting. Also, the device gives the opportunity to set haptic vibration feedback after 15 min or 30 min of consecutive sitting time. The Activator is the successor of the SitFIT, which was developed in the EuroFIT intervention trial [16]. The Activator has no longer a screen and USB-port, making the Activator more water-resistant and decreasing the production costs. Participants will be encouraged to wear the device as frequent as possible to obtain the most accurate results.

In addition to the Activator, participants will receive a 12-week self-help sit less, move more program booklet. This sit less, move more program is intended to set weekly graded goals to ultimately increase physical activity with 4500 steps and upright time with 60 min. This sit less, move more program is intended especially for those who sit more than 65% of waking time and those that take less than 10.000 steps a day. Intervention targets were based on our experiences in the EuroFIT lifestyle intervention [15]. In the self-help program booklet, participants are encouraged to: 1) self-monitor and record daily steps, upright time and percentage sitting time; 2) make specific weekly steps and upright time goals involving detailed planning of where, when, how and with whom; 3) identify barriers and find solutions (relapse prevention); and 4) weekly review of previously set behavioural goals and intentions. In the booklet simple messages around behaviour-health information, information on consequences, and practical tips on how to incorporate small changes into daily life are also provided.

### Data collection

Data will be collected at three time points: baseline, approximately 3 months and 12 months after baseline assessment. Table 1 presents a summary of all measurements conducted in the study. Data will be collected by a trained researcher team. Questionnaire data will be collected on a tablet redirecting participants to an online questionnaire format (Collector, Survalyzer). In order to promote retention, all participants who completed 12 months measurements will receive a small incentive. Primary outcome of the trial is the difference between the intervention and control condition in objectively assessed daily sitting time at 12 month follow-up. Secondary outcomes of the trial at short-term (≈3 months) and long-term follow up (≈12 months) comprise:

- Daily sitting time (3 months follow up)
- Daily standing time, stepping time, steps, number of bouts > 30 min, and sit-to-stand transitions
- Occupational sitting time, standing time, stepping time, steps, number of bouts > 30 min, and sit-to-stand transitions
- Work-related outcomes (e.g. work absenteeism, work presenteeism, work performance, work-related stress, desk-based worktime, transportation to work)
- Self-reported lifestyle behaviour (e.g. sleeping time, smoking, self-reported sedentary behaviour)
- Health-related outcomes (e.g. Body mass index (BMI), waist circumference, musculoskeletal symptoms, need for recovery, vitality)

**Table 1:** Overview of study outcomes and measurement instruments used

	Baseline	3 months	12 months
Objectively measured sitting, upright time and step count			
ActivPAL^tm^ micro	x	x	x
ActivPAL wearing diary (sleep, work time)	x	x	x
Self-reported lifestyle behaviour			
Sedentary behaviour (WSQ)	x	x	x
Sleeping time	x	x	x
Smoking status	x	x	x
Transport mode to and from work	x	x	x
Co-interventions	x	x	x
Work-related outcomes			
Work performance (IWPQ)	x	x	x
Work-related stress (ERI-S)	x	x	x
Work content and desk based worktime	x	x	x
Work absenteeism (self-reported by iPCQ and company records)		x	x
Work presenteeism (self-reported iPCQ)		x	x
Health-related outcomes			
Body weight	x	x	x
Body height	x		
Waist circumference	x	x	x
Musculoskeletal symptoms (SNQ)	x	x	x
Need for recovery (NFR)	x	x	x
Vitality (Vita-16© TNO)	x	x	x
Quality of life (EQ-5D-5 L)	x	x	x
Healthcare utilisation (self-reported by iMCQ and insurance records)		x	x
Self-reported socio demographics			
Age	x		
Gender	x		
Marital status	x		
Number of children	x		
Household composition	x		
Education	x		
Household income	x		
Employment status (including length of tenure, job classification, working hours per week, overtime work, days working from home)	x		
Postal code	x		

#### Objectively measured sitting, upright time and steps

Sitting and upright time and step counts will be assessed using the activPAL^tm^ micro (PAL Technologies Ltd., Glasgow, UK) activity monitor, which is preferably worn on the right front thigh for seven consecutive full days. The activPAL has been found to have good measurement properties to assess sitting, standing, and stepping time in adults [17–19]. It is a small device (9 g) and does not give feedback to the wearer. Participants are asked to wear the device continuously, 24/7 with a nine-day protocol and only temporarily remove the device during swimming or bathing activities. First and last day will be removed for analyses. Participants will also complete a short log diary to indicate the exact wearing time (e.g. time put on and taken off), sleeping time (e.g. time went to bed and time got up), and occupational time (e.g. time started work and time finished work). ActivPAL data will be considered valid if participants wore the device more than 20 h per day for at least four days [20].

#### Self-reported lifestyle behaviours

Self-reported sedentary behaviour will be assessed by using the Workforce Sitting Questionnaire (WSQ), which has acceptable measurement properties to measure sitting at work and to measure total sitting time [21]. In this questionnaire participants are asked to report the time spent sitting in hours and minutes (1) while travelling to and from places; (2) while at work; (3) while watching TV; (4) while using a computer at home; and (5) while doing other leisure activities on both a usual workday and a non-workday in the past seven days.

Sleeping time is measured with a single item question in which participants are asked to report the time spend sleeping on average in the last seven days. Smoking behaviour will be measured, including date of quitting and amount of current consumption, when relevant. Usual mode of transport to and from work will be assessed with the following answer options: private vehicle, public transportation, bicycle, by foot, a combination of public transport and bicycle use, or other. Distance to work will be based on residence postal code to company postal code.

#### Work-related outcomes

Individual work performance will be measured using the Individual Work Performance Questionnaire (IWPQ), which has shown to be a valid and reliable instrument [22–24]. Two scales of the IWPQ will be used: task performance (5 items) and contextual performance (8 items). Participants are asked to reflect on the past three months and answer the questions on a five-point scale from 0 (‘seldom’) to 4 (‘always’) for task performance and contextual performance. A mean score for each subscale is calculated by adding the item scores and dividing their sum by the number of items in the subscale.

The imbalance between efforts and rewards with regard to work will be measured with the self-reported Effort-Reward Imbalance Short (ERI-S) questionnaire [25], which has been found to be a reliable questionnaire [26]. The ERI-S consists of 16 four-point Likert scale questions (strongly disagree (1) - strongly agree (4)), in which participants are asked to indicate to what extent the items reflect their typical work situation in their present occupation. The questionnaire consists of three psychometric scales: effort (three questions), reward (seven questions), and overcommitment (six questions). The reward subscale is further specified in self-esteem (two questions), promotion (three questions), and security (two questions). Higher ratings in the effort scale relate to higher efforts, whereas lower ratings in the reward scale relate to lower rewards.

Work content during a usual working day will be measured by a single question whereby participants need to divide a total of hundred percent over computer work, meetings, multimedia use, breaks, or other tasks. Worktime spent at the desk on a usual working day (i.e. < 4 h, 4–6 h, 6–8 h, > 8 h) and longest uninterrupted period spent sitting behind the desk (i.e. < 30 min, 30–60 min, 1–1.5 h, 1.5–2 h, > 2 h) will be recorded.

#### Health related outcomes

Body weight will be assessed at all measurements using calibrated electronic flat scale (SECA 877, SECA, Burmingham, UK) with accuracy to the nearest 0.1 kg. Participants will be allowed to wear normal clothes, but will be asked to remove any heavy items of clothing (e.g. jackets), their shoes and any items in their pockets. Body height will be measured only at baseline after participants have removed their shoes using a portable stadiometer (SECA 206, SECA, Birmingham, UK) with accuracy to the nearest millimetre. BMI will be calculated as weight in kilograms divided by the square of height in meters (kg/m^2^).

Waist circumference will be measured twice at the level midway between the lowest ribcage and the iliac crest using a non-expendable tape (SECA 201, SECA, Birmingham, UK) with accuracy to the nearest 0.1 cm. Participants will be asked to remove their shirts. If the first two waist measurements differ by ≥0.5 cm, a third measurement will be done. The mean circumference will be calculated from the (two nearest) measures.

Musculoskeletal symptoms will be assessed based on the modified Nordic Musculoskeletal questionnaire (SNQ) [27]. Participants are asked if they experienced any pain or discomfort in the following anatomical regions: 1) neck, shoulders or upper back; 2) arms, wrists or hands; 3) lower back; 4) hips, thighs, knees, ankles or feet, in the past three months (no; yes, sometimes; yes, regularly; yes, prolonged). If participants answer yes, they are asked to indicate the maximum intensity of the worst symptoms on an eleven-point scale (‘0’ no pain – ‘10’ extremely painful).

Difficulties workers experience in recovering from work-related exertion will be assessed by using the Need For Recovery scale (NFR) [28]. The NFR comprises of eleven items with dichotomous answering categories (yes/no). A score is calculated by adding all the scores of the individual items and transforming these into a scale from 0 to 100. Higher scores indicate a higher need for recovery after work. The NFR has shown favourable test-retest reliability and sensitivity to detect change [29].

Vitality will be assessed by using the Vita-16© TNO questionnaire [30], which consists of the following three core dimensions: energy, motivation and resilience. Energy is characterized by feeling energized and full of pep, motivation is defined by setting goals in life and putting effort into achieving these goals, and resilience is characterized by the ability to cope with daily life problems and challenges. These three dimensions are measured with a 16-item questionnaire on a seven-point Likert scale (‘1’ hardly to ‘7’ always), which has been found valid and reliable in a Dutch adult population [30].

Quality of Life will be assessed with the EQ-5D-5 L questionnaire [31, 32]. Participants are asked to rate their mobility, self-care, usual activities, pain/discomfort, and anxiety/depression on a five-point scale (i.e. no problems, slight problems, moderate problems, severe problems, or extreme problems). They are also asked to rate their today’s health on a scale ranging from zero (worst health imaginable) to one hundred (best health imaginable).

#### Socio-demographic characteristics

At baseline, age, gender, ethnicity, marital status, number of children living in their household, household composition, education level, household income, employment status (i.e. length of tenure, working hours, job classification, overtime hours and number of days working from home), postal code and possible co-interventions (e.g. participation in other lifestyle or health programs, access and use of sit-stand desks or cycling workstations) will be assessed.

### Economic evaluation

Work absenteeism and presenteeism will be self-reported and measured using iMTA Productivity Cost Questionnaire (iPCQ) [33] at three and twelve months. The iPCQ questionnaire measures on both time points sick leave and productivity losses while being at work but not functioning optimally. Work absenteeism will also be assessed using company records.

Healthcare utilization will be assessed using an adapted version of the iMTA Medical Cost Questionnaire (iMCQ) at three and twelve months. This questionnaire will include the number of visits to the general practitioner, allied health professionals or complementary healthcare providers, medication use, the number of ambulatory visits at a hospital or other health care organizations, and admissions to a hospital or other health care organizations. Healthcare utilization will also be assessed using company records for those with insurance at the insurance company.

The costs for the delivery of the program will be calculated using a bottom-up approach using costs reported by the insurance company (i.e. preparation, coordination and administration, recruitment of participants, implementation and delivery of the program, and materials including Activator, sit-stand desks, cycling workstations and office balls), and the participating university (i.e. preparation and start-up, and materials).

Dutch standard costs will be used to value health care utilization and productivity losses [34] . Patient and family costs other than informal care will be valued using self-reported prices. Medication costs will be estimated using prices of the Royal Dutch Society for Pharmacy. For the valuation of absenteeism from paid work, the friction cost approach will be used.

### Process evaluation

An extensive process evaluation is embedded in this study to investigate processes that are necessary for sustainable implementation of the Dynamic Work intervention, the way in which the intervention affects outcomes, and facilitators and barriers for adoption, implementation and continuation. The mixed-method design of this process evaluation has been informed by MRC guidance on process evaluation of complex interventions [35] and builds on previous extensive process evaluations [16, 36, 37]. Data will be gathered from participants, heads of department/team managers, occupational physiotherapists, and Dynamic Work coordinators. Researchers will keep field notes in which relevant information is reported of phone calls, email and measurement observations. Furthermore, usage data from the Activator app will be collected remotely (e.g. logs of data upload and error reports). Table 2 presents an overview of the process evaluation objectives and methods used ordered in the domains context, implementation and mechanism of impact.

**Table 2:** Overview of process evaluation objectives and methods used

	Q-B	Q-3M	Q-12M	FG-3M	INT-w	Activator	AR	Logbook	INT-OP- 12M	FG-HD/TM-12M	INT-DWc-12M	FN
CONTEXT												
Reach												
Characteristics of participating departments (e.g. size, facilities and work content).								X		X	X	X
Characteristics of occupational physiotherapist (e.g. background, demographics, and experience).									X			
Characteristics of participants (e.g. demographics and health risk profile).	X											
Facilitating factors and barriers to delivery of Dynamic Work at departments												
Perceived barriers and facilitators for implementation, attractiveness to participate, successes and failures regarding recruitment and intervention delivery, intention for continuation, and future activities.									X	X	X	
IMPLEMENTATION												
Recruitment												
Sources and procedures for recruitment of departments, and reported reasons for (non-)participation.										X	X	X
Sources and procedures for recruitment of occupational physiotherapists to deliver Dynamic Work in participating departments.									X		X	X
Sources and procedures for recruitment of participants, and reported reasons for (non-)participation and withdrawal from the study.	X			X	X					X	X	X
Delivery												
Total number of installed sit-stand desks and cycling workstations, number of delivered plenary and on-site counselling sessions, number of occupational physiotherapist consultations, and number of distributed Activators and self-help program booklets (dose delivered).						X		X			X	X
Participation in the Dynamic Work intervention (e.g. number of attended sessions, coach consultations and use of sit-stand desk, cycling workstation, Activator and self-help program booklet) (dose received).		X	X	X		X	X	X				
The extent to which occupational physiotherapists delivered the face-to-face session with the head of the department/team managers, the plenary Dynamic Work sessions and on-site department counselling sessions as intended (fidelity).								X				
MECHANISMS OF IMPACT												
Experiences of the Dynamic Work intervention and which elements were most useful												
Head of department/team manager and coordinator views and experiences of adopting and implementing the Dynamic Work intervention.										X	X	X
Occupational physiotherapists’ experience with the Dynamic Work intervention and view on essential elements in supporting participants to make behavioural change.									X			X
Participants’ experiences and perceived effectiveness with the Dynamic Work intervention, and their view on essential elements in supporting them to make behavioural changes.		X	X	X								X

*Abbreviations*: *Q-B* Questionnaire participant baseline, *Q-3M* Questionnaire participant 3M, *Q12-M* Questionnaire participant 12M, *FG-3M* Focusgroup participant 3M, *INT-w* Interview withdrawal participant, *AR* Attendance record, *INT-OP-12M* Interview occupational physiotherapist 12M, *FG-HD/TM-12M* Focusgroup head of department/ team manager 12M, *INT-DWc-12W* Interview Dynamic Work coordinator, *FN* field notes reseracher

At baseline participants will be asked how they were attracted to participate in the Dynamic Work intervention and what their reasons were for participation. At follow-up assessments, intervention participants will be asked about their experiences with the Dynamic Work intervention, dose received, perceived effect of the program, and support. Reasons for withdrawal or dropout from the study (measurements) will be registered. Around 3 months after the start of the intervention, from each department 6–8 intervention participants will be invited to participate in a focus group interview to understand: reasons for joining the Dynamic Work intervention, experiences with the Dynamic Work intervention, the impact it has had on their life, their views on essential program elements for making behavioural change, and their suggestions for improvement of the intervention.

At 12 months, all intervention condition heads of departments and team managers will be invited to join a focus group session to gather opinions and experiences with delivery of the Dynamic Work intervention, experiences with recruiting participants for the Dynamic Work study, and barriers and facilitators for adoption, implementation and continuation.

The occupational physiotherapists will be asked to complete attendance and online logs after each team meeting and each on-site department counselling session. The log contains preparation and delivery time, a 10-point scale to score the session, questions on delivery of the intervention, and open-ended questions in terms of what worked and what did not work. At 12 months, all occupational physiotherapists will also be invited for an in-depth interview to gather demographic information, their experiences with (delivering) the Dynamic Work intervention, their view on the essential elements for support in making behavioural change, facilitators and barriers for adoption, implementation and continuation, and their suggestions for changes to the intervention.

Semi-structured interviews will be conducted with the two Dynamic Work coordinators to gather barriers and facilitators for adoption, implementation and continuation, and experiences in recruiting departments for the trial.

Trained researchers (not in direct contact with the occupational physiotherapists, team managers or Dynamic Work coordinators) will use semi-structured interview guides for conducting aforementioned focus groups and interviews. All interviews and focus groups will be audiotaped and fully transcribed verbatim and anonymized.

### Sample size and power calculation

A minimal statistically detectable difference in the primary outcome of 45 min change in total sitting time was chosen. Standard deviations and intraclass correlation (ICC) were based on the results of a previous study in the occupational setting that aimed to reduce sitting time and also used the activPAL as primary outcome measure [10]. To correct for clustering effects within departments and expecting an average of 20 participants per cluster we took an ICC of 0.021 (design effect of 1.40) into account. A standard deviation (SD) of 80.7 was used, with a power of 90%, and alpha of 0.05. Hence, when taking a dropout of 25% into account, sample size of 250 included participants is needed.

### Data handling

All collected data will be kept strictly confidential. All participants will be given a unique ID number, not by name (apart from the consent form). All data will be kept in a secure location within the Amsterdam UMC, location VUmc, accessible only by members of the research team during the active phase of the study; afterwards all data will be archived in line with the policy of VUmc. At the end of the study (i.e. after all final measurements) all participant will receive a digital report of their results (e.g. objectively measured sitting time, standing time, steps, weight, height, and waist circumference) with appropriate explanation. Our university has an internal procedure for auditing ongoing trials for which the current study could be selected. A data monitoring committee is not needed given the fact the intervention is considered low risk. In the unlikely case an adverse event is reported by a participant the principal investigator will be informed. If any adverse event is deemed to be a serious adverse event after consultation with the principal investigator this is reported to the VUmc Medical Ethic Committee within 24 h.

### Statistical analyses

A detailed Statistical Analysis Plan will be developed, and finalized prior to database lock. Data analyses will be conducted after completion of the data collection at 12 months, no interim analysis are planned. Consistent with the cluster-randomized design, multilevel linear regression analyses will be performed according to the intention-to-treat principle with the primary outcome at 12 months as the dependent variable, while study group allocation and baseline value of the primary outcome will be modelled as independent variables. Secondary outcomes will be analysed in a similar way using multilevel linear or logistic regression analyses. Analyses will be performed with multilevel analysis for Windows (MLwiN version 2.22; Centre for Multilevel Modelling, University of Bristol, Bristol, UK) and SPSS version 22 (IBM Corp, Armonk, New York, USA). Statistical significance will be set at *p* < 0.05.

To evaluate the cost-effectiveness of the intervention program statistical analyses will be also done according to the intention-to-treat principle. Missing cost and effect data will be imputed using multiple imputation according to the MICE algorithm developed by van Buuren [38]. Rubin’s rules will be used to pool the results from the different multiply imputed datasets. Bivariate regression analyses will be used to estimate cost and effect differences between intervention and comparison group while adjusting for confounders if necessary. Incremental Cost-Effectiveness Ratios (ICERs) will be calculated by dividing differences in costs between the conditions by the differences in clinical outcomes and Quality-Adjusted Life-Years (QALYs). Clinical outcomes that will be included in the cost-effectiveness analyses are: changes in sitting time, upright time, step counts, BMI, waist circumference, and self-reported physical health. Bias-corrected and accelerated bootstrapping with 5000 replications will be used to estimate 95% confidence intervals around the cost differences and statistical uncertainty surrounding the ICERs. Uncertainty surrounding ICERs will be graphically presented on cost-effectiveness planes. Cost-effectiveness acceptability curves will also be estimated showing the probability that the intervention program is cost-effective in comparison with usual practice for a range of different ceiling ratios thereby showing decision uncertainty [39].

To evaluate the process data of this study descriptive statistics (mean, SD, proportions) will be used to report participants’, occupation physiotherapists’ and departments’ characteristics and results of pre-structured questions from the questionnaires, attendance sheets and coach logs. Descriptive statistics and visualizations will also be used to explore and report the use of the Activator. All reported (suggestions and reasons for) adaptations to the program and any other answers to open-ended questions will be listed, analysed and summarized. A framework analysis approach, following the TCID-framework of Flottorp et al. (2013) [40], will be used to identify barriers and facilitators for adoption, implementation and continuation. Further, we will calculate an ‘implementation score’ by combining specific selected process items reported by participants on delivery or/and dose of the intervention (rated on a five-point Likert scale) [41]. Computing this score will allow us to define low and high implementers and explore differences between departments.

## Discussion

This paper describes the design of a cluster randomized study that will evaluate the multicomponent Dynamic Work intervention in desk-based office workers. The Dynamic Work intervention builds on previous developmental work of the participating insurance company and previous experiences of the research team in occupational health interventions focused on reducing sitting through the utilization of sit-stand workstations [36, 42, 43], as well as activity/sitting trackers [16]. Our work and that of others has shown that sit-stand desks are a promising environmental intervention to reduce occupational sitting time at least in the short-term [7, 9] and possibly also in the longer term [10]. The current trial will, to our knowledge, be the first to combine sit-stand desks (and other activity permissive workstations) with an activity tracker that allows for real time feedback of sitting behaviour. Together with the coaching components of the intervention this makes it a unique occupational health intervention that aims to reduce sitting time during as well as outside working hours in the longer-term.

Through the current trial an extensive evaluation of the Dynamic Work intervention will take place. This evaluation will identify changes in objectively assessed sitting and upright time, and step counts, intermediate health outcomes as well as work-related outcomes and will include robust economic and process evaluations. The work-related outcomes and economic evaluation from the company perspective will be of direct relevance for employers, as a cost-effective intervention will have the potential for wider implementation and roll out in other companies. The detailed process evaluation will evaluate mechanisms of impact and will help to identify the strengths and weakness of the Dynamic Work intervention. These will be used to formulate recommendations for future improvement and refinement of the intervention, which will be essential in the light of potential wider implementation and roll out.

In conclusion, the current cluster randomized controlled trial will assess whether the multicomponent Dynamic Work intervention is effective in reducing sitting time in desk-based office workers in the longer-term as compared to usual practice. In addition, using a company and societal perspective, we will provide insight into the cost-effectiveness of the Dynamic Work intervention as compared to usual practice. It will also be assessed whether the reduction in sitting time is related to a range of key health and work-related outcomes, and how the Dynamic Work intervention can possibly be further improved.

Date initial submission to BMC Public Health: 4 May 2017.

## Abbreviations

BMI: Body mass index
ERI-S: Effort-Reward Imbalance Short
FG: Focus group
FTE: Full time equivalent hours
ICC: Intraclass correlation
ICER: Incremental Cost-Effectiveness Ratios
iMCQ: iMTA Medical Cost Questionnaire
INT: Interview
iPCQ: iMTA Productivity Cost Questionnaire
IWPQ: Individual Work Performance Questionnaire
NFR: Need For Recovery scale
Q: Questionnaire
QALY: Quality-Adjusted Life-Years
SD: Standard deviation
SNQ: Nordic Musculoskeletal questionnaire
WHO: World Health Organisation
WSQ: Workforce Sitting Questionnaire
